# PACAP38 Differentially Effects Genes and CRMP2 Protein Expression in Ischemic Core and Penumbra Regions of Permanent Middle Cerebral Artery Occlusion Model Mice Brain

**DOI:** 10.3390/ijms150917014

**Published:** 2014-09-23

**Authors:** Motohide Hori, Tomoya Nakamachi, Junko Shibato, Randeep Rakwal, Masachi Tsuchida, Seiji Shioda, Satoshi Numazawa

**Affiliations:** 1 Division of Toxicology, Department of Pharmacology, Toxicology and Therapeutics, School of Pharmacy, Showa University, 1-5-8 Hatanodai, Shinagawa, Tokyo 142-8555, Japan; E-Mails: author3cityform@yahoo.co.jp (M.H.); numazawa@pharm.showa-u.ac.jp (S.N.); 2 Department of Anatomy, School of Medicine, Showa University, 1-5-8 Hatanodai, Shinagawa, Tokyo 142-8555, Japan; E-Mails: nakamachi@med.showa-u.ac.jp (T.N.); rjunko@nifty.com (J.S.); datsucchie@hotmail.co.jp (M.T.); 3 Laboratory of Regulatory Biology, Graduate School of Science and Engineering, University of Toyama, Toyama 930-8555, Japan; 4 Laboratory of Exercise Biochemistry and Neuroendocrinology, Institute of Health and Sports Sciences, University of Tsukuba, 1-1-1 Tennoudai, Tsukuba, Ibaraki 305-8574, Japan; 5 Organization for Educational Initiatives, University of Tsukuba, 1-1-1 Tennoudai, Tsukuba, Ibaraki 305-8577, Japan

**Keywords:** ischemic core, penumbra, PACAP38, differential gene expression, *Gabra6*, *S100a5*, *Il6*, *Tph2*, *Prlr*, CRMP2

## Abstract

Pituitary adenylate-cyclase activating polypeptide (PACAP) has neuroprotective and axonal guidance functions, but the mechanisms behind such actions remain unclear. Previously we examined effects of PACAP (PACAP38, 1 pmol) injection intracerebroventrically in a mouse model of permanent middle cerebral artery occlusion (PMCAO) along with control saline (0.9% NaCl) injection. Transcriptomic and proteomic approaches using ischemic (ipsilateral) brain hemisphere revealed differentially regulated genes and proteins by PACAP38 at 6 and 24 h post-treatment. However, as the ischemic hemisphere consisted of infarct core, penumbra, and non-ischemic regions, specificity of expression and localization of these identified molecular factors remained incomplete. This led us to devise a new experimental strategy wherein, ischemic core and penumbra were carefully sampled and compared to the corresponding contralateral (healthy) core and penumbra regions at 6 and 24 h post PACAP38 or saline injections. Both reverse transcription-polymerase chain reaction (RT-PCR) and Western blotting were used to examine targeted gene expressions and the collapsin response mediator protein 2 (CRMP2) protein profiles, respectively. Clear differences in expression of genes and CRMP2 protein abundance and degradation product/short isoform was observed between ischemic core and penumbra and also compared to the contralateral healthy tissues after PACAP38 or saline treatment. Results indicate the importance of region-specific analyses to further identify, localize and functionally analyse target molecular factors for clarifying the neuroprotective function of PACAP38.

## 1. Introduction

Our group has been investigating a medical condition of the brain, namely brain ischemia also known as cerebral ischemia/ischemic stroke that is regarded as the third most common cause of death after heart attack and cancer [1], and the potential neuroprotective effect of pituitary adenylate-cyclase activating polypeptide (PACAP) therein [2,3,4,5,6,7,8,9,10,11]. Utilizing the established permanent middle cerebral artery occlusion (hereafter referred to as PMCAO) model mice, our experimental strategy was based on first providing an inventory of the molecular factors, genes and proteins that are differentially expressed in the ischemic brain hemispheres with or without PACAP38 treatment [12,13,14]. To note, these studies employed the intraluminal filament technique PMCAO model because of its simplicity and noninvasive characteristics compared to the classical electrocoagulation method for PMCAO. The transient MCAO results in reperfusion injury, and to avoid this complication, the method of choice in our research projects was using the intraluminal PMCAO model.

Applying the DNA microarray (4x44K whole genome transcriptomic profiling) and two-dimensional gel electrophoresis (2-DGE) linked with mass spectrometry approaches [15,16,17], our previous research revealed the first ischemia-related transcriptome and proteome of the mouse brain, laying a strong foundation for studies designed to elucidate the mechanisms regulating ischemia and to explore the neuroprotective effects of agents such as target neuropeptides, including PACAP38 [12,13,14]. The large-scale transcriptomic data obtained from these above studies are available under the accession/series numbers GSE 28201 and GSE 37565 at the NCBI Gene Expression Omnibus (GEO) public functional genomics data repository (http://www.ncbi.nlm.nih.gov/geo/info/linking.html). Readers are referred to these gene expression profiles for further information, download, and/or independent analysis.

**Figure 1:**
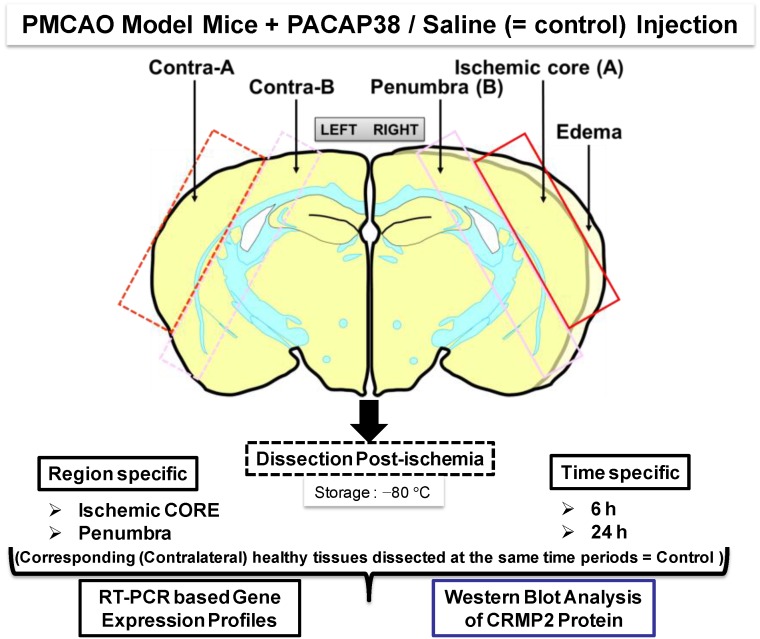
Experimental design and workflow using the permanent middle cerebral artery occlusion (PMCAO) model. Effect of the intracerebroventricular administered pituitary adenylate-cyclase activating polypeptide 38 (PACAP38) into ischemic mouse brain was evaluated at the molecular level in the ischemic core and penumbra of the ipsilateral (**right**) hemisphere along with the corresponding regions in the healthy contralateral hemisphere (**left**). All marked brain regions were sampled and finely powdered in liquid nitrogen, followed by investigation into molecular level changes at the level of genes (mRNA abundance) and protein expression by reverse transcription-polymerase chain reaction (RT-PCR) and Western blotting, respectively.

Though these were novel findings at the level of transcriptome and proteome providing an overall picture of the ischemic hemisphere at two time points of 6 and 24 h post-ischemia, the specificity of expression and localization of these identified molecular factors in infarct core and penumbra (ipsilateral), and matching non-ischemic regions (contralateral) remained incomplete. With this in mind, we asked a question—is there any discernable difference between different regions of the ischemic brain? To answer that, our present study targeted ischemic core and penumbra regions in the ischemic hemisphere comparing it to the corresponding contralateral (healthy) core and penumbra regions at 6 and 24 h, post-PACAP38 or -saline injections. The same molecular factors, genes and proteins that were identified in our previous studies [12,13] were examined. The experimental strategy used for this study is presented in the Figure 1 (see below).

To test our hypothesis that there would be certain differences in expression of these gene and proteins, depending on the brain regions (core and penumbra) examined, we performed (I) reverse transcription-polymerase chain reaction (RT-PCR) analysis of selected genes; and (II) Western blot analysis of the collapsin response mediator protein 2 (CRMP2). The CRMP2, first named as CRMP-62, was originally identified in 1995 using a *Xenopus laevis* oocyte expression system [18]. CRMPs, being multifunctional adaptor proteins/microtubule associated proteins, are highly expressed in brain/central nervous system [19,20]. In particular, CRMP2 is known to be important in determination of neuronal polarity and axonal elongation, involved in axonal damage and neuronal cell death, neurological disorders, and brain ischemia among other functions being gradually revealed [21,22,23,24,25,26,27]. One conclusion drawn from these past and recent studies is providing a proof-of-concept on pharmacological manipulation of CRMP2 as a possible therapy development against certain neurological diseases [28,29].

The results presented and discussed below revealed not only distinct expression profiles of these genes and the CRMP2 protein in ischemic core and penumbra, but also provided some new data over the previous two studies where the brain hemispheres were used. These results are primarily discussed in this communication, and the importance of targeted brain region analysis is one of the key messages of this paper, with regard to further understanding and clarifying the neuroprotective action of PACAP38.

## 2. Results

Figure 1 illustrates post-PMCAO, the brain regions labeled ischemic core and penumbra in the ipsilateral (ischemic) hemisphere that were dissected out carefully using a sterile scalpel. In parallel, similar sized regions in the contralateral hemisphere were also dissected. Both PACAP38 injected and saline injected brains were dissected and sampled with extreme care at 6 and 24 h post-ischemia and immediately frozen at −80 °C. It is to be noted that the ischemic core and penumbra was demarcated based on the visual confirmation and past experience and observations post-triphenyltetrazolium chloride (TTC) staining of ischemic brains.

**Figure 2:**
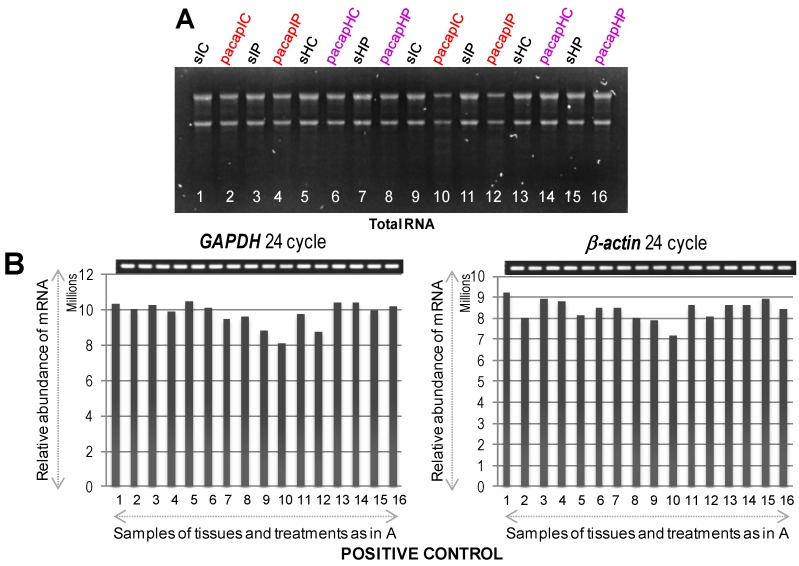
Total RNA visualized by agarose gel electrophoresis (**A**) followed by mRNA expression profiles of *GAPDH* and *β-actin* genes used a positive control (**B**) to confirm equal loading and proper cDNA synthesis. In (**A**), lane numbers **1** to **8** and **9** to **16** indicate 6 and 24 h samples (1/9 = sIC, saline ischemic core; 2/10 = pacapIC, PACAP38 ischemic core; 3/11 = sIP, saline Ischemic Penumbra; 4/12 = pacapIP, PACAP38 ischemic penumbra; 5/13 = sHC, saline healthy core; 6/14 = pacapHC, PACAP38 healthy core; 7/15 = sHP, saline healthy penumbra; 8/16 = pacapHP, PACAP38 healthy penumbra), respectively; and in (**B**), gel images on top show PCR product bands stained with ethidium bromide; band intensities are also presented graphically below for clarity.

**Figure 3:**
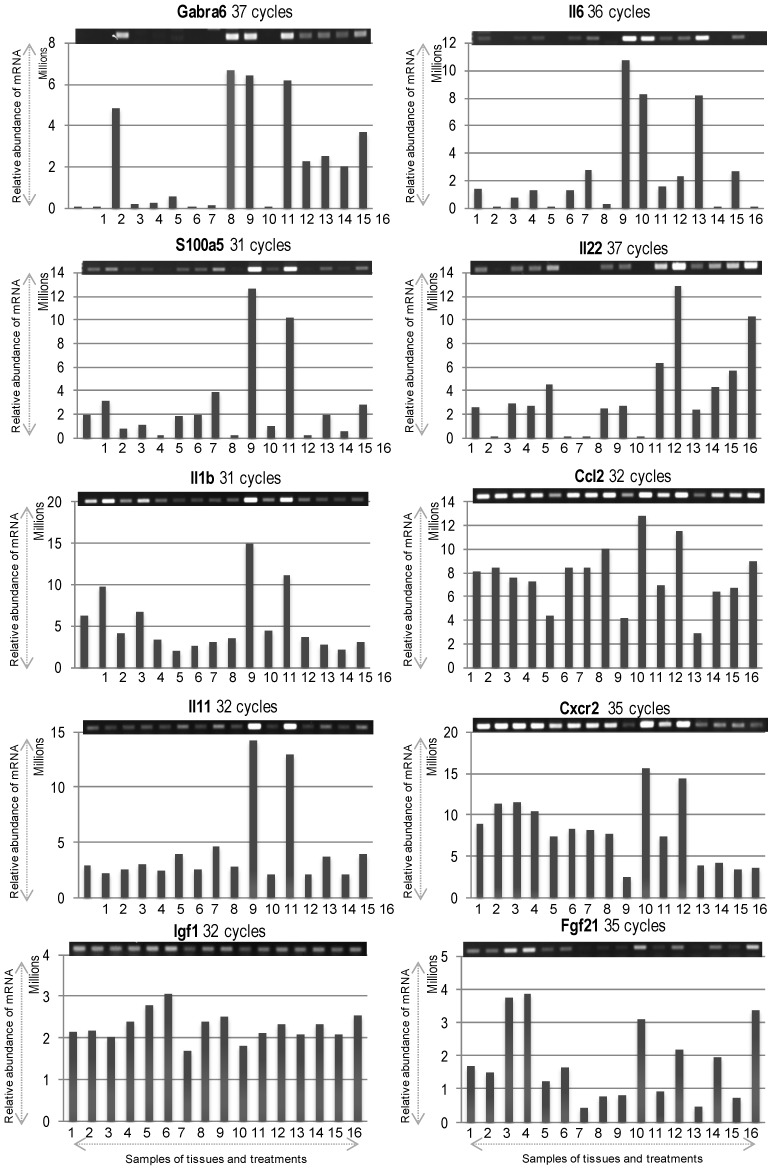
The mRNA expression profiles of differentially expressed up-regulated genes. Gel images on top show PCR product bands stained with ethidium bromide; band intensities are also presented graphically below for clarity. Lane numbers **1** to **8** and **9** to **16** indicate the 6 and 24 h samples (**1**/**9** = sIC, saline Ischemic Core; **2**/**10** = pacapIC, PACAP38 Ischemic Core; **3**/**11** = sIP, saline Ischemic Penumbra; **4**/**12** = pacapIP, PACAP38 Ischemic Penumbra; **5**/**13** = sHC, saline Healthy Core; **6**/**14** = pacapHC, PACAP38 Healthy Core; **7**/**15** = sHP, saline Healthy Penumbra; **8**/**16** = pacapHP, PACAP38 Healthy Penumbra), respectively. Semi-quantitative RT-PCR was performed as described in Materials and Methods, and the gene-specific primers are detailed in Table 1.

**Figure 4:**
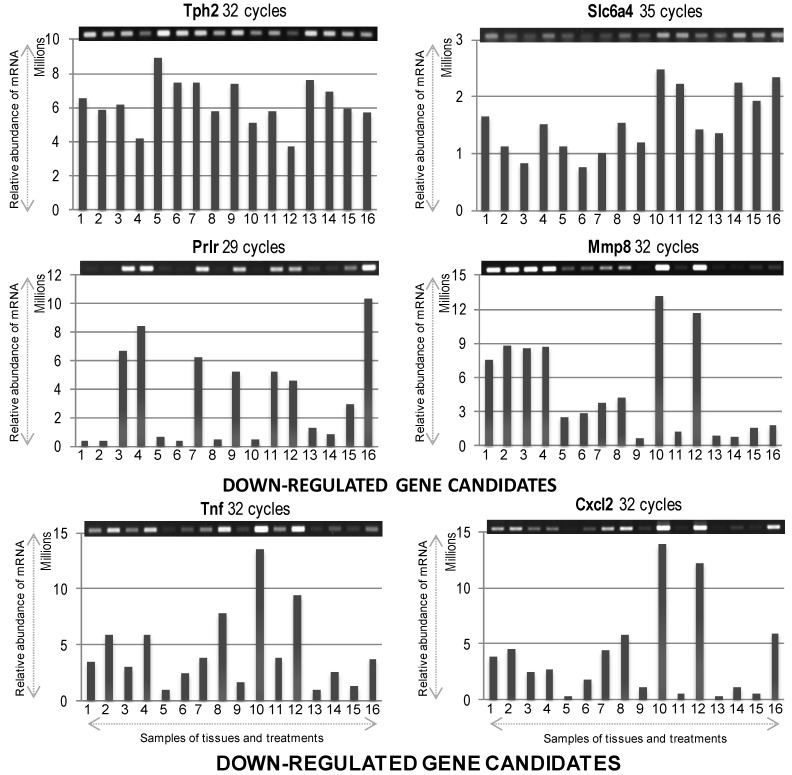
The mRNA expression profiles of differentially expressed down-regulated genes. Gel images on top show PCR product bands stained with ethidium bromide; band intensities are also presented graphically below for clarity. Lane numbers **1** to **8** and **9** to **16** indicate the 6 and 24 h samples (**1**/**9** = sIC, saline Ischemic Core; **2**/**10** = pacapIC, PACAP38 Ischemic Core; **3**/**11** = sIP, saline Ischemic Penumbra; **4**/**12** = pacapIP, PACAP38 Ischemic Penumbra; **5**/**13** = sHC, saline Healthy Core; **6**/**14** = pacapHC, PACAP38 Healthy Core; **7**/**15** = sHP, saline Healthy Penumbra; **8**/**16** = pacapHP, PACAP38 Healthy Penumbra), respectively. Semi-quantitative RT-PCR was performed as described in Materials and Methods, and the gene-specific primers are detailed in Table 1.

At first, we used these designated brain regions for extraction of total RNA confirming their appropriate quantity and quality (Figure 2A). As shown in Figure 2B, the expression of *glyceraldehyde 3-phosphate dehydrogenase* (*GAPDH*) and *β-actin*, as positive controls, was confirmed in all dissected regions from both hemispheres at 6 and 24 h by RT-PCR. Semi-quantitative RT-PCR was performed as described in Materials and Methods, and the gene-specific primers are detailed in Table 1. Results showed that the mRNAs for *GAPDH* and *β-actin* were expressed almost uniformly across the tested conditions. With these confirmatory data, we proceeded to check the mRNA expression profiles of selected target genes (Table 1), which were selected based on our previous research on PACAP induced or suppressed genes under ischemic conditions [12,13]. Both up-regulated (see below, Figure 3) and down-regulated (see below, Figure 4) genes were selected for testing the hypothesis that there would be differences in the expression level of these genes depending not only on the regions (ischemic core and penumbra) but also the time post-PMCAO. We describe each expression below. In general, almost all genes examined showed an induction and suppression as originally seen in the Hori *et al.* (2012) study [13].

**Table 1:** Primer combinations used for RT-PCR.

Accession (Gene)	Forward Primer		Reverse Primer		Product Size (bp)	Description
	Primer Name	Nucleotide Sequence (5'-3')	Primer Name	Nucleotide Sequence (5'-3')		
NM_001001303	MS099	gctacactgaggaccaggttgt	MS100	ctcctgttattatgggggtctg	306	*GAPDH*
NM_007393	MS101	acgttgacatccgtaaagacct	MS102	ggtgtaaaacgcagctcagtaa	302	*β-actin*
NM_008068	MS495	cttcaccaatctccagtcacag	MS496	gcaaaagctactgggaagagaa	312	*Gabra6*
NM_031168	MS245	agaggataccactcccaacaga	MS246	ctgaaggactctggctttgtct	322	*Il6*
NM_011312	MS235	gctgaccctgagtaggaaagaa	MS236	ataggaggggcagttaaagagg	264	*S100a5*
NM_016971	MS497	actgttgacacttgtgcgatct	MS498	ctgactcctcggaacagtttct	276	*Il22*
NM_008361	MS499	tgacgttcccattagacaactg	MS500	tcaaactccactttgctcttga	342	*Il1b*
AK050118	MS501	tggttcaaactaaccattgcag	MS502	ggagcttccccttcagtatttt	261	*Igf1*
NM_011333	MS505	ttttgtcaccaagctcaagaga	MS506	attaaggcatcacagtccgagt	279	*Ccl2*
NM_020013	MS519	ctctctatggatcgcctcactt	MS520	tgtaaaggctctaccatgctca	277	*Fgf21*
NM_008350	MS703	ggcctgctgttgttaaagactc	MS704	atctcagttccctgctcttcag	284	*Il11*
NM_009909	MS699	tcgtcttcagcaaacacctcta	MS700	ctgatgggtaggaggcagtatc	330	*Cxcr2*
NM_013693	MS171	tactcccaggttctcttcaagg	MS172	gctgggtagagaatggatgaac	330	*Tnf*
NM_010484	MS507	ccctctgtttctcctgttcatc	MS508	atgtcctctccctcagtgtgtt	279	*Slc6a4*
NM_173391	MS509	cgtatggagcagggttactttc	MS510	tcctgcaccacattctcaatac	283	*Tph2*
NM_008611	MS241	ggttaggatgagccataagtgc	MS242	atgtgcttgagaaaggaatggt	273	*Mmp8*
NM_011169	MS491	ggagttcttggaagtggatgac	MS492	tgtatgacaattgggggtgtta	334	*Prlr*
NM_009140	MS521	aacatccagagcttgagtgtga	MS522	ataacaacatctgggcaatgg	338	*Cxcl2*

### 2.1. Upregulated Genes

*Gabra6* (*γ-aminobutyric acid (GABA) A receptor 6*) gene was highly induced in penumbra under saline injection at 6 h, and at 24 h in ischemic core the gene was more abundantly expressed after both saline and PACAP38 treatment. However, in penumbra, *Gabra6* was only induced after PACAP38 treatment. In contralateral hemisphere, its expression was seen at a lower level only at 24 h.

*Il6* (*interleukin 6*), showed a strong expression at 24 h compared to 6 h, predominantly in ischemic core after both saline and PACAP38 injection. A higher expression was also seen in healthy core after saline treatment. Overall, a higher expression level was seen at 24 h post-ischemia.

*S100a5* (*S100 calcium binding protein A5*), showed the strongest expression at 24 h in PACAP38 treated ischemic core and penumbra samples.

*Il22* (*interleukin 22*) gene showed a greater overall induction at 24 h post-ischemia, in penumbra after both saline and PACAP38 treatments. Moreover, it showed an increased expression in healthy penumbra, especially under PACAP38 treatment compared to the saline treatment.

*Il1b* (*interleukin-1β*) gene similarly showed strongest expression at 24 h after PACAP38 injection in both ischemic core and penumbra. A similar trend in expression was also seen at 6 h post-ischemia.

*Ccl2* (*chemokine C-C motif ligand 2*), a chemotactic chemokine, in general showed a constitutive expression over all samples, however, its mRNA abundance was strongest at 24 h after PACAP38 injection in both ischemic core and penumbra.

*Il11* (*interleukin 11*) gene showed strongest expression at 24 h after PACAP38 treatment in both ischemic core and penumbra compared to a similar constitutive expression in all other samples.

*Cxcr2* (chemokine receptor type 2) gene showed an almost high constitutive expression at 6 h, but was highly induced at 24 h after PACAP38 injection in both ischemic core and penumbra.

*Igf1* (*insulin-like growth factor 1*), which is a eurotrophic and differentiation factor playing key role in regulation of growth and development of central nervous system (CNS) revealed an almost high constitutive expression at 6 and 24 h. Looking at *Igf1* mRNA abundance, it can be seen that this gene did not show a strong increase after PACAP treatment when compared to the other PACAP38 highly up-regulated genes presented in Figure 3. This might be due to the highly constitutive baseline expression of *Igf1* in the brain. In the above context, it should be noted that at 6 h time point, a slight increase in abundance was seen in healthy controls, especially with PACAP38 treatment. Looking back at gene expression data from previously performed brain hemisphere DNA microarray analysis [12,13]; *Igf1* was constitutively expressed at high level, but at 6 h after PACAP38 treatment under ischemic condition showed higher mRNA abundance compared to all other time points and treatments.

*Fgf21* (*fibroblast growth factor-21*) is a new member of FGF (fibroblast growth factor) super-family and an important endogenous regulator of glucose and lipid metabolism, but less investigated in CNS. Its expression was strongly induced at 6 h under saline and PACAP38 treatment in ischemic penumbra. Its mRNA abundance was also seen at 24 h after PACAP38 injection in both ischemic core and penumbra. More interestingly, PACAP38 alone induced its expression in healthy core and penumbra at 24 h.

### 2.2. Down-Expressed Genes

*Tph2* (*tryptophan hydroxylase 2*) mRNA expression despite showing a higher baseline or constitutive expression in all samples, showed a slight decrease in abundance after PACAP38 treatment at both 6 and 24 h, especially prominent in ischemic penumbra. This was also similar to observed down-regulation of transcript abundance in the brain hemisphere in our previous studies [12,13]. The notable difference was that compared to the previous study, here we could identify the decrease at both 6 and 24 h in the penumbra, whereas in the Hori *et al.* (2012) study, the decrease was only seen at 6 h, albeit in the hemisphere [13].

*Slc6a4* (*solute carrier family 6 member 4*), encoding for the neurotransmitter transporter, serotonin, gene expression was also constitutively high in all samples analyzed. However, the 6 h samples showed a lower expression compared to 24 h, which was also observed in the Hori *et al.* (2012) study [13]. In particular, ischemic core and healthy core after PACAP38 treatment showed slightly decreased mRNA expression.

*Prlr* (*prolactin receptor*) gene also showed suppressed expression in both healthy core and penumbra (strongest decrease) after PACAP38 treatment at 6 h. However, 24 h after PACAP38 injection strongly reduced the *Prlr* expression in ischemic core, and also to some degree in ischemic penumbra and healthy core.

*Mmp8* (*matrix metalloproteinase 8*) gene did not show a clear reduction in transcript abundance, except for a slight suppression seen at 24 h in healthy core with PACAP38 treatment. However, its abundance was drastically lower in healthy regions compared to ischemic regions at both 6 and 24 h.

*Tnf* (*tumor necrosis factor*) gene also did not show a reduction in its transcript by PACAP38 treatment, although, its abundance was lower in the saline ischemic and healthy core regions, and in the saline injected healthy penumbra.

*Cxcl2* (*chemokine C-X-C motif ligand 2*) gene also did not show any reduction in transcript abundance after PACAP38 treatment, and behaved in a similar fashion as the *Tnf* gene. It should be noted that in case of the last three down-regulated genes (*Mmp8*, *Tnf*, and *Cxcl2*) compared to the saline treated core and penumbra, where all three genes were strongly down-regulated, a strong increase in mRNA abundance was seen in both ischemic core and penumbra at 24 h after PACAP38 treatment.

### 2.3. Collapsin Response Mediator Protein 2 (CRMP2) Abundance

**Figure 5:**
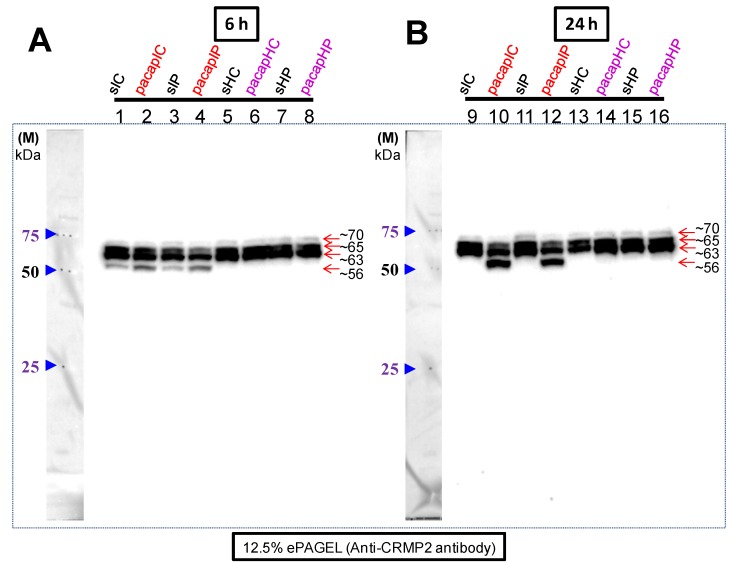
Western blot analysis of the CRMP2 protein cross-reacting proteins in ischemic core and penumbra of ipsilateral (**right**) hemisphere along with their abundance in corresponding regions in the healthy contralateral hemisphere (**left**). In (**A**), lane numbers **1** to **8** and in (**B**), lane numbers **9** to **16** indicate the 6 and 24 h samples, respectively. Abbreviations are the same as in Figure 2 legend. Proteins cross-reacting with the anti-CRMP2 protein antibody are visible as three constitutively present proteins (~70, ~65, and ~63 kDa size) in all samples. The ~56 kDa cross-reacting protein is seen not only in the ischemic core but also in the penumbra. Total protein extraction, separation, Western blotting, and image analyses procedures are detailed in Materials and Methods.

In our previous study we had observed cross-reaction of the CRMP2 antibody with a newly appeared ~56 kDa protein band only in the PMCAO model [13]. In this study, using the same specific brain regions examined for gene express changes, we found that there was a weak expression of the ~56 kDa CRMP2 cross-reacting protein band in both the ischemic core and penumbra at 6 h, but whose intensity was stronger under PACAP38 treatment (Figure 5A,B). At 24 h, PACAP38 injection dramatically increased its (~56 kDa) abundance in both the ischemic core (strongest increase) and penumbra (weaker than that seen in the core region). No such cross-reaction with a band of similar size was seen in the healthy core and penumbra at 6 and 24 h in either PACAP38 or saline treatment. On the other hand, with respect to ~65 kDa and in particular ~63 kDa CRMP2 cross-reacting protein bands, their abundance was high in healthy core and penumbra, where the bands were strongly increased over the ischemic regions. However, the difference in these two CRMP2 cross-reacting bands was the most prominent at 24 h, where on the one hand in the ischemic regions (lanes 10 and 12) their intensity decreased but on the other hand in the healthy tissues (lanes 14 and 16) their abundance increased with PACAP38 treatment. Although, weakly cross-reacting, the 70 kDa band appeared to show a similar trend.

## 3. Discussion

The main objective addressed in this study was to identify PACAP38 influenced gene expression and CRMP2 protein abundance, specifically in the ischemic core and the penumbra as compared to the first two studies carried out using whole hemispheres [12,13]. Results on both gene expression and the CRMP2 protein indicate that expression patterns for these molecules vary in different brain regions in the PACAP38 treated ischemic hemisphere compared to healthy contralateral regions including saline treatment (Figure 3, Figure 4 and Figure 5). Thus, it would be important to apply such an experimental design to future experiments involving brain ischemia studies. Gene expression analysis of these selected genes using RT-PCR also provided new information into the localized expression in the ischemic core and penumbra. We further discuss below some of the gene expression data obtained here focusing on observed differences in expression between infract core and penumbra in relation to PACAP38 treatment.

Among the highly expressed genes with no defined function in neuroprotection two genes are worth mentioning. First, *Gabra6*, whose link with neuroprotective mechanism remains undiscovered, was strongly induced at 24 h not only in ischemic core under both saline and PACAP38 injections but also in ischemic penumbra by only PACAP38 injection. These results suggest that penumbra might be a site where PACAP38 is actively affecting *Gabra6* transcription and that 24 h might be a critical time period for asserting such action. Although, we could not a detect *Gabra6* transcript in ischemic penumbra under saline, at 24 h, its mRNA abundance was higher compared to the 6 h time point, even in healthy core and penumbra. We cannot explain this result, but speculate that healthy tissues might be a site for enhanced *Gabra6* expression in response to the ischemia or as a bilateral effect. Second, expression profile of the *S100a5* gene revealed its dramatic expression at 24 h compared to 6 h, and PACAP38 injection increased its expression at all treatment points, and most strongly in the ischemic core and penumbra. This suggests that the effect of *S100a5* is critical at the 24 h time period and site of action is both ischemic core and penumbra. However, to date the role of S100a5 protein in neuroprotection remains unknown.

Among the cytokines, which play an important role in mediating the inflammatory responses in the ischemic brain, especially Il6, we found that not only Il6 expression but also the transcripts of *Il22*, *Il1b*, and *Il11* were differentially expressed in these brain regions. The *Il6* gene expression was found to be the most strongly induced in the ischemic core rather than penumbra at 24 h, and also in the corresponding healthy core regions. As the 24 h time period presents its (*Il6*) strongest expression, this result is in line with the fact that IL6 is an important inflammatory cytokine in ischemic brain [6]. However, at 24 h, PACAP38 was found to suppress its expression not only in the ischemic core, and also in the healthy core and penumbra. This suggests that IL6, which is also believed to have a role in neurodegeneration rather than neuroprotection in cerebral ischemia [30], is responsive to PACAP38 treatment, which might be one of the mechanisms by which PACAP38 could exert a neuroprotective role in the brain during ischemia. In the case of *IL22*, its expression was strongly induced by PACAP38 treatment at 24 h in both the ischemic and healthy penumbras but that was especially high in the ischemic penumbra. Although not much is known about the biological role of this cytokine in neuronal cells, a recent study using naïve PC12 cells suggested a role for IL22 in neurological processes, a first such report showing a neuroprotective function for this cytokine [31]. Another unrelated work on lung infection by the influenza A virus showed that IL22 limits lung inflammation and subsequent bacterial superinfections [32]. These two examples might explain the possible function of *Il22* in the PACAP38 influenced neuroprotection in the ischemic brain. Looking at another cytokine, *Il1b*, which showed a different expression pattern where PACAP38 induced its transcript in the ischemic core followed by ischemic penumbra that was strongest at 24 h. Similarly, the *Il11* expression was strongest at 24 h, in the ischemic core followed by ischemic penumbra. Both *Il1b* and *Il11* have been suggested to have a negative role in brain injury or neuroprotection. For example, neutralizing IL-1b could reduce cerebral edema and tissue loss causing an improvement in late cognitive outcome following traumatic brain injury in mice [33]. In case of *IL11*, a cold pre-conditioning neuroprotection is dependent on TNF-α (tumor necrosis factor α) but was enhanced by blocking IL11 [34]. The potential neuroprotective function of these two cytokines in ischemic brain post-PACAP38 treatment remains to be investigated.

Looking at the chemokines, *Ccl2* and *Cxcr2*, we found that unlike the cytokines, these are quite highly expressed constitutively in all samples, especially at 6 h in the case of the *Cxcr2* gene. Despite this high expression, at 24 h, both these genes were strongly expressed in PACAP38 treated ischemic core followed by penumbra. The CCL2 is an important mediator of neuroinflammation mediating macrophage recruitment and migration during peripheral and central nervous system inflammation [35]. CXCR2, a major chemokine receptor, and its ligands, have been implicated in numerous brain pathologies, and are involved in the recruitment of neutrophils and developmental positioning of neural cells [36,37]. It is interesting to note that the both *Ccl2* and *Cxcr2* genes are being induced by PACAP38 in the ischemic core and penumbra. How PACAP38-mediated neuroprotection is linked to the induction of these pro- and anti-inflammatory chemokines and their receptors in the ischemic brain remains to be decisively answered in future studies.

For the down-regulated genes, we discuss two unique genes that encode for serotonin and prolactin receptor. *Tph2*, tryptophan hydroxylase 2, encodes a protein responsible for synthesizing the neurotransmitter serotonin (5-hydroxytryptamine; 5-HT); 5-HT is synthesized in two steps, with TPH (tryptophan hydroxylase) as the rate-limiting enzyme [38]. Results revealed that the baseline expression of *Tph2* in all samples was high, indicating that the transcript is abundantly present under the performed experimental conditions. Nevertheless, PACAP38 treatment caused a slight decrease in expression of *Tph2* transcript abundance, especially in the ischemic penumbra at both 6 and 24 h. Although, the decrease is small, it would be tempting to link this observed decrease in *Tph2* gene expression to changes in the level of serotonin; but, we have no data to support that claim as yet. There are reports in the literature showing the decrease in 5-HT level following neonatal hypoxia-ischemia [39]. Moreover, a recent report shows that NAS, *N*-acetylserotonin that is an immediate precursor of melatonin, has neuroprotective function against ischemic injury [40]. We are working to establish a protocol for their (metabolites) measurement by tandem mass spectrometry, and after which it may be possible to shed more light on our observations at the level of mRNA expression. The second gene encoding for a receptor of a hormone involved in human reproductive health, namely *Prlr*, prolactin receptor, showed a strong decrease in its expression upon PACAP38 treatment in the ischemic core at 6 and 24 h. Our data also showed increased expression of *Prlr* at both 6 and 24 h in the ischemic penumbra. This difference might be explained by a previously published report showing for the first time that hypoxia ischemia induces a robust activation of the prolactin axis in regions of the cerebral cortex affected by injury [41]. However as described in that report, and we quote the authors—“The lack of neuroprotective properties *in vivo* and *in vitro* indicates that, unlike growth hormone, prolactin is not directly involved in neuronal rescue in the injured brain. Its strong relation to glial reactions and its gliatrophic effects suggest that the prolactin axis is primarily involved in a gliogenic response during recovery from cerebral injury [41]”. Looking at the other down-regulated gene expressions analyzed in this study, we could not find clear differences in the suppression by PACAP38 in analyzed core and penumbra tissue samples, although these genes were selected based on our previous microarray data using whole ischemic hemispheres [12,13,14]. For example, in the case of *Mmp8*, *Tnf*, and *Cxcl2* down-regulated genes they showed higher expression after PACAP38 treatment in core and penumbra at 24 h over saline controls. Although, we cannot explain the discrepancy, it is possible that the observed gene expression results are due to the use of specific sample tissues—Core and penumbra.

Finally, we examined the behavior of the CRMP2 (CRMP family member) [18,19,20,21,22,23] protein abundance and expression in the core and penumbra after PACAP38 treatment. With respect to the results obtained from CRMP2 Western blotting, there was a difference (quantitative) in the results obtained using the brain hemispheres [13] and the brain regions (ischemic core and penumbra) as shown in Figure 5. In our previous study, at 6 h after PACAP38 treatment, the most notable result was of the identification of the ~56 kDa protein band, which at that time we believed to be strongly induced in the ischemic hemisphere, whereas at 24 h, at best a very weak expression could be seen [13]. Additionally, in that paper, immunofluorescence staining suggested that the penumbra also has a positive reaction to the CRMP2 antibody at 6 h, which is consistent with the findings of this study showing the CRMP2 cross-reacting ~56 kDa band in penumbra regions at 6 h PACAP38 treatment (Figure 5). In the present study, we have clarified that at 24 h post-ischemia the ~56 kDa CRMP2 cross-reacting protein band strongly correlated with PACAP38 treatment, especially in the core regions. Initially, two questions arise from this observation- first, why is there a lower ~56 kDa band and second, how is it produced? One previously published study, specifically on the CRMP2 protein, has suggested that a 55 kDa band (which should be the same to our observed 56 kDa protein, Figure 5) is a degradative product following both acute traumatic and neurotoxic injury; those authors also stated, and we quote “Further studies are being conducted to elucidate the pathophysiological significance of the cleavage of CRMP-2 in the nervous system” [25]. If this is also the case in our study and, which the study of Zhang *et al.* (2007) suggests [25], then why is there enhanced CRMP2 degradation (as evident by the ~56 kDa band) after PACAP38 treatment? Searching the literature, we found another study demonstrating that a short isoform of the CRMP2 protein (around 58 kDa) derived from post-translational *C*-terminal processing of the CRMP2B subtype has an important role in regulation of neurite outgrowth in brain development [42]. That study suggests that our observed ~56 kDa might not simply be a degradative product, but on the contrary might be specifically targeted for such processing after PACAP38 treatment.

We further turned our attention to the mature (unphosphorylated) CRMP2 protein that has a molecular weight of around 62 kDa, and the phosphorylated form whose molecular weight is around 66 kDa [21]. Although in the ischemic regions (lanes 1 to 4 and 9 to 12) the ~63 and ~66 kDa proteins decrease after PACAP38 treatment, in the contralateral side (healthy regions) there is a strong increase in abundance of especially the ~63 kDa band at 6 and 24 h. One possible reason may be that PACAP38 directly affects the CRMP2 expression in healthy contralateral hemisphere, which in turn regulates several signaling pathways in promoting nervous system development, as proposed earlier [42]. Based on all these results, there is no doubt to the observation that the CRMP2 protein expression and abundance is influenced by PACAP38. However, the question remains as to how PACAP38 neuroprotective function can be linked to the established role of full-length CRMP2 in axonal growth [20,21,22,43]. Our data showing a reduction in the ~63 and ~66 kDa bands implies that there might be a stop to un-regulated axonal growth and other functions related to nervous system development in the ischemic regions, which may be expected as the brain tissues, especially the core that is dying. Simultaneously, an increase in the short isoform of ~56 kDa could be associated with a specific function of causing neurite outgrowth inhibition and suppressed axonal growth in the ischemic regions, as supported by the 2008 study of Rogemond and co-workers using neuroblastoma cells and cultured cortical neurons [42]. Nevertheless, whether CRMP2 stability has a role in PACAP-mediated neuroprotection or is just a reflection of the ischemic brain must be clarified.

Currently, we have initiated a new project to examine in detail the CRMP2 protein, by (I) using an immunoprecipitation approach for identifying interacting proteins; (II) re-examining its phosphorylation state using specific antibodies; and (III) examining the presence of splice variants using exon-PCR/exon microarray. The idea is that all these additional experiments will shed new light on CRMP2 function under PACAP38 treatment. It would also be worth investigating how other members of the CRMP family differ in expression in these brain regions with or without PACAP.

## 4. Materials and Methods

### 4.1. Animals and Husbandry

Animal care and experimental procedures were used as approved by the Institutional Animal Care and Use Committee of Showa University (School of Medicine), Tokyo, Japan. Thirty male mice (C57BL/6J) of 9-weeks-old (25~35 g body weight) were purchased from Charles River (Kanagawa, Japan). Mice were housed at the Animal Institution in Showa University in acrylic cages (8 mice/cage) maintained at 23 °C with a standard 12 h light/dark cycle, optimum humidity, and temperature control. Animals were given access to tap water and laboratory chow *ad libitum*.

### 4.2. Permanent Middle Cerebral Artery Occlusion, PACAP38 and Saline Treatments

The experimental design is presented in Figure 1. The PMCAO model mice were generated as described previously [12,13]. Mice were first anesthetized with 4% sevoflurane (induction) and 2% sevoflurane (maintenance) in a 30% O_2_ and 70% N_2_O gas mixture via a face mask, followed by an incision in the cervical skin, opening of salivary gland, resulting in visualization of the right common carotid artery. A midline cervical incision was made to expose the external carotid artery, and using intraluminal filament technique the PMCAO model was generated. PACAP38 (1 µL containing 1 pmol) or 1 µL of saline (0.9% NaCl, as control) was injected intracerebroventrically, immediately after PMCAO. PACAP38 (Peptide Institute Inc., Osaka, Japan; supplier temperature was −20 °C) was dissolved at 10^−5^ M concentration by saline, and stored at −80 °C. PACAP test solution (for injection) was diluted ×10 times with 0.9% NaCl just before use. After injections, the animals were returned to their cages. A total of four groups were prepared as mentioned in Table 2.

In the present study, we used three mice each in PMCAO groups for PACAP38 and saline injections, respectively, that exhibited neurological grades G1 and G2 [6,12,13] for the subsequent downstream analysis. The neurological grades were based on a previously published paper in our group by Ohtaki and co-workers [6]. Briefly, evaluation of the neurological deficits was based on five grades 0 to 4: (I) 0 represents—normal, no observable neurological deficit; (II) 1 represents—mild, failure to extend the contralateral forepaw upon lifting the animal by the tail; (III) 2 represents—moderate, circling to the contralateral side; (IV) 3 represents—severe, leaning to the contralateral side when at rest; and (V) 4 represents—heavy, no spontaneous motor activity [6]. Some of the mice were examined for ischemia by TTC staining of brain sections (2 mm slices) at 37 °C for 10 min [6,12].

### 4.3. Dissection of Brain, Sampling and Storage

Six or 24 h post-injection of PACAP38 or saline, the mice were removed from their cages, decapitated, and their brains carefully removed on ice. The right (ipsilateral; ischemic) and left (contralateral) hemispheres were dissected, and from each hemisphere the ischemic core and penumbra regions and corresponding healthy core and penumbra were carefully removed with a sterile scalpel, and placed in 2 mL Eppendorf tubes. The samples were then quickly immersed in liquid nitrogen and stored in −80 °C prior to further analysis (Figure 1).

**Table 2:** The number of mice used for this experiment. A total of 23 mice were prepared, and 3 each were selected randomly based on neurological grade (NG). Mice with score 0 and 3 were not selected for sampling the brains.

Group 1: PMCAO Injected Normal Saline, and Decapitated after 6 h		
Mouse Tail No.	Body Weight Before Operation (g)	Neurological Grade (NG)
1	22.87	2
2	22.53	3
6	25.97	2
7	23.47	2
9	24.43	0
10	23.15	2
11	22.21	0
12	23	2
**Group 2: PMCAO Injected Normal Saline, and Decapitated after 24 h.**		
Mouse Tail No.	Body Weight Before Operation (g)	Neurological Grade (NG)
2	24.15	1
3	22.55	0
4	22.82	1
5	23.64	2
6	22.51	0
4 *	23.05	3
5 *	24.44	2
6 *	23.87	2
7 *	23.54	3
9 *	23.6	3
**Group 3: PMCAO Injected PACAP38, and Decapitated after 6 h.**		
Mouse Tail No.	Body Weight Before Operation (g)	Neurological Grade (NG)
1	24.57	1
4	25.17	1
5	23.89	2
**Group 4: PMCAO Injected PACAP38, and Decapitated after 24 h.**		
Mouse Tail No.	Body Weight Before Operation (g)	Neurological Grade (NG)
7	23	2
9	23.29	3
10	23.91	2

*, indicates dead mice.

### 4.4. Total RNA Extraction, Synthesis of cDNA and Reverse Transcription-Polymerase Chain Reaction

Stored brain regions were ground to a very fine powder with liquid nitrogen, and the total RNA was extracted whose quantity and quality was determined and confirmed exactly as described previously [12,13]. Three samples were pooled for each condition as the amount of each region was very small, and the resulting powder was used for total RNA extraction. For validation of total RNA quality and subsequently synthesized cDNA, RT-PCR was carried out using two commonly used house-keeping genes *GAPDH* and *β-actin* as positive controls [12,13]. The gene-specific primers were designed (Table 1). The cDNA synthesis and RT-PCR analysis protocol used is as follows: Total RNA samples were first DNase-treated with RNase-free DNase (Stratagene, Agilent Technologies, La Jolla, CA, USA). First-strand cDNA was then synthesized in a 20 μL reaction mixture with an AffinityScript QPCR cDNA Synthesis kit (Stratagene, Agilent Technologies, La Jolla, CA, USA) according to the protocol provided by the manufacturer, using 1 μg total RNA. The reaction conditions were: 25 °C for 5 min, 42 °C for 5 min, 55 °C for 40 min and 95 °C for 5 min. The synthesized cDNA was made up to a volume of 50 μL with sterile water supplied in the kit. The reaction mixture contained 0.6 μL of the first-strand cDNA, 7 pmols of each primer set and 6.0 μL of the Emerald Amp PCR Master Mix (2× premix) (TaKaRa Shuzo, Shiga, Japan) in a total volume of 12 μL. Thermal-cycling (Applied Biosystems, Tokyo, Japan) parameters were as follows: after an initial denaturation at 97 °C for 5 min, samples were subjected to a cycling regime of 20 to 40 cycles at 95 °C for 45 s, 55 °C for 45 s, and 72 °C for 1 min. At the end of the final cycle, an additional extension step was carried out for 10 min at 72 °C. After completion of the PCR the total reaction mixture was spun down and mixed (3 μL), before being loaded into the wells of a 1.2/1.8% agarose (Agarose (fine powder) Cat no. 02468-95, Nacalai Tesque, Kyoto, Japan) gel. Electrophoresis was then performed for ~22 min at 100 Volts in 1× TAE (tris-acetate) buffer using a Mupid-ex electrophoresis system (ADVANCE, Tokyo, Japan). The gels were stained (8 μL of 10 mg/mL ethidium bromide in 200 mL 1× TAE buffer) for ~7 min and the stained bands were visualized with the ChemiDoc XRS+ imaging system (Bio-Rad, Hercules, CA, USA). Each gene expression analysis was performed at least twice as independent PCR reactions and electrophoresis on gel, and one of the images was presented as a representative data for each gene in the respective figures for up-/down-regulated expressions.

### 4.5. Extraction of Total Soluble Protein

Total protein was extracted from sample powders (around 50 mg) of each dissected brain region using a previously used lysis buffer containing thiourea and Tris (LB-TT) for extraction of brain proteins [13,16,17]. The composition of slightly modified LB-TT was as follows: 7 M (*w*/*v*) urea, 42 g; 2 M (*w*/*v*) thiourea, 15.2 g; 4% (*w*/*v*) CHAPS (3-[(3-cholamidopropyl) dimethylammonio]-1-propanesulfonate), 4.0 g; 18 mM (*w*/*v*) Tris–HCl (pH 8.0), 1.8 mL; 14 mM (*w*/*v*) trizma base, 169.5 mg; 0.2% (*v*/*v*) triton X-100, 0.2 mL; 50 mM (*w*/*v*) DTT (dithiothreitol), 771.5 mg; 1% (*v*/*v*) pH 3–10 ampholyte, 1 mL; and two EDTA-free proteinase inhibitor (Roche Diagnostics GmbH, Mannheim, Germany) tablets in a total volume of 100 mL at room temperature (RT). To extract protein, 1 mL of LB-TT was quickly added to the 2 mL microfuge tube containing the sample powder (immediately after removal from the −80 °C deep freezer) and immediately mixed by vortexing (at full speed using a Lab mixer, Iwaki, Tokyo, Japan) for 1 min at RT. The protein solution in LB-TT was incubated at RT for 30 min with mixing by vortexing (for 30 s) and sonication (for 30 s in a water-bath type sonicator) for a total of 5 times. The insoluble protein pellet and/or debris were pelleted by centrifugation at 18,500× *g* for 15 min at 20 °C in a high-speed refrigerated micro centrifuge (MX-150, TOMY, Tokyo, Japan). The clear supernatant (around 900 µL) was transferred to a new 1.5 mL microfuge tube, and stored at –80 °C as the total soluble protein. Protein concentration was determined with a Coomassie Plus™ Protein Assay kit (PIERCE, Rockford, IL, USA) using bovine serum albumin (BSA) as a standard and a NanoDrop 2000 spectrophotometer (Thermo Scientific, Wilmington, DE, USA).

### 4.6. Western Blot Analysis

The separated proteins after SDS-PAGE (mini-gel) were transferred onto a polyvinyldifluoride (PVDF) (Trans-Blot Turbo Midi PVDF, 0.2 µM, Transfer Packs kit; Cat. no. 170-4157, Bio-Rad, Hercules, CA, USA). The Trans-Blot Turbo Transfer System (Bio-Rad, Hercules, CA, USA) was used for the electrotransfer (Standard SD protocol; 25 V, 1.0 A, 30 min). Following the transfer of proteins on the PVDF membrane (and also confirmed by visualizing all the 10 colored molecular mass standards), it was incubated in 25 mL of 5% blocking solution (Block-Ace powder, Cat. no. UK-B80, DS Pharma, Osaka, Japan; Yukizirushi, Sapporo, Hokkaido, Japan) for 1 h under constant slow shaking at RT. Blocking solution was prepared by dissolving 4 g powder in 80 mL 1× TTBS (tris-tween-buffer-saline) (10× TTBS: NaCl, 80 g; 1 M Tris–HCl, pH 7.5, 200 mL; Tween-20, 5 mL). Western blotting and detection was carried out using the Immun-Star WesternC Chemiluminescent kit (Cat. no. 170-5070, Bio-Rad, Hercules, CA, USA) following the manufacturer’s instructions. Blocking solution was decanted and the membrane was washed once in 1× TTBS (5 min), followed by incubation in 25 mL of primary antibody solution (PAS; 1 µL rabbit anti-CRMP2 protein antibody (Cat. no. ab62661; 100 µg, 2 mg/mL; Abcam, Cambridge, UK, www.abcam.co.jp)) for 1 h, as above. The membrane was then washed with 25 mL of 1× TTBS for five times. The last TTBS wash was decanted and the membrane was incubated in 25 mL of secondary antibody solution (SAS; 0.5 µL of Amersham, ECL (enhanced chemiluminescence) anti-rabbit IgG, HRP (horseradish peroxidase) linked species-specific whole antibody (from Donkey); Cat. no. NA 934; GE Healthcare, Little Chalfont, Buckinghamshire, UK) for 1 h with slow shaking at RT. The 1× TTBS wash step was repeated five times. For blot development, the luminol/enhancer and peroxide buffer solutions were mixed in a 1:1 ratio (1 mL:1 mL; one membrane volume) and spread over the membrane and incubated at RT for 5 min. Excess solution was drained by touching one end of the membrane on a KimWipe paper towel, and the signal (cross-reacting protein bands) was visualized using the ChemiDoc XRS+ imaging system (Bio-Rad, Hercules, CA, USA). The Western blot analysis was repeated at least three times, and representative data from one blot are shown.

## 5. Conclusions

Having described the above, it should be noted that without using or dissecting out the core and penumbra regions in the ischemic brain, the present results on gene and protein expression would not have been realized. Thus, the importance of studying, as far as possible, different regions of the brain in greater detail at the level of the gene and protein is necessary. Based on both our previous [12,13,14] and the current data we are a step closer to understanding the gene expression changes in specific regions of the brain, and which might serve as a base for exploring their translation in clinical studies in context of observed PACAP-mediated neuroprotection in an animal model. In the meantime such studies can be considered and performed, the next step in this research is whole genome (DNA microarray) analysis of the ischemic core and penumbra tissue in conjunction with additional bioinformatics analysis using the Ingenuity Pathway Analysis (IPA, Ingenuity^®^ Systems, 1700 Seaport Blvd, 3rd Floor Redwood City, CA 94063, USA) tool, and co-immunoprecipitation experiments utilizing the anti-CRMP2 antibodies. These experiments have been initiated following our present results discussed above, and it is our belief that identifying specific molecular pathways genome-wide along with clarifying role of the ~56 kDa CRMP2 protein will help in understanding how PACAP38 might function as a neuroprotective and/or potentially therapeutic agent in counteracting ischemia.
